# Predictors of Serum Total IgE in a Random Sample of 7–17 Year Old Children

**DOI:** 10.5402/2011/169859

**Published:** 2011-05-31

**Authors:** Sofie Strømgaard, Simon Francis Thomsen, Mogens Fenger, Vibeke Backer

**Affiliations:** ^1^Department of Respiratory Medicine, Bispebjerg Hospital, 2400 Copenhagen, Denmark; ^2^Department of Dermatology, Bispebjerg Hospital, 2400 Copenhagen, Denmark; ^3^Department of Clinical Biochemistry, Hvidovre Hospital, 2650 Hvidovre, Denmark

## Abstract

*Background*. There is little knowledge of the causes for raised serum total immunoglobulin E (IgE) in children. We investigated the association between perinatal, socioeconomic, demographic, clinical, paraclinical, and early life factors, and total serum IgE. *Methods*. In a random sample of 7–17 year old children, data on possible risk factors were collected using a questionnaire and total serum IgE was measured in venous blood. Airway responsiveness to inhaled histamine was measured and skin prick tests were performed using common aeroallergens. Data was analysed using linear regression. *Results*. Positive skin prick test (*P* < .001), airway hyperresponsiveness (*P* = .003), atopic dermatitis (*P* = .046), and parental predisposition to allergy (*P* = .047) showed a significant relation to serum total IgE levels. There was no association of smoking to serum total IgE. *Conclusions*. This study showed a strong positive relationship between markers of the atopic syndrome and serum total IgE levels in children. In contrast to some earlier studies, we did not find a significant relation between smoking status and IgE.

## 1. Introduction

There is only limited knowledge about factors that cause increased serum total immunoglobulin E (IgE) in children. Generally, IgE has been found to be higher in men than in women [1, 2]. However, some studies found this difference only among smokers [3, 4], whereas others have found that the difference was confined to the age group above 55 years [5, 6]. Serum total IgE has been shown to decrease with age with the highest concentrations found in children and adolescents [2, 5, 7]. Some investigations have found this association only in different subgroups of the study populations but results are contradictory [3, 4, 6, 8]. The association between allergic symptoms and serum total IgE has been found to be dependent on atopic status [4, 9, 10]. Notably, subjects with positive skin prick test have higher IgE levels [4, 7]. Rhinitis, wheeze, and current asthma are closely associated with IgE [10]. Furthermore, subjects with occupational exposure to dust or gas have been shown to have higher IgE levels than those not exposed [6]. Most of these studies were on adult populations, whereas some also included children above the age of 15. One study included children older than 11 years and three included children older than 6 years. In one-year olds, IgE has been found to be associated with parental IgE levels [11], whereas some studies have found an effect of alcohol intake on serum total IgE in adults [12]. Smoking has been shown to increase IgE in both men and women [1, 2, 6, 9] but especially in men [3, 8]. Warren et al. found that smoking was associated with increased IgE only in men [8], whereas another study found that the association between smoking and increased IgE was present only in subjects older than 20 years of age [4]. Due to the relatively sparse knowledge of predictors of serum total IgE in children we studied the association between perinatal, demographic, socioeconomic, clinical, paraclinical and early life factors, and serum total IgE in a population sample of children aged 7–17 years. 

## 2. Methods

### 2.1. Subjects

A random sample of children and adolescents living in Copenhagen, Denmark was invited to take part in a clinical examination [13]. All subjects were drawn at random from the civil registration list. The mean age was 12.1 years (age range 7–17 years, SD = 2.9 years). A total of 480 subjects (response rate, 33.3%) participated in the survey of whom 222 (46.3%) were males. To examine the representativeness of the examined population, telephone interviews were conducted among 116 randomly selected subjects from the group of nonrespondents. The subjects, interviewed by telephone, did not differ significantly from the subjects originally included with respect to sex, age, or prevalence of asthma, but there were significantly fewer children with symptoms of hay fever among the group of nonrespondents. 

### 2.2. Interview and Questionnaire

Information on possible risk factors was collected by means of a parental multi-item questionnaire. The questions were answered in the participating families' homes prior to testing and in cases of doubt consensus was reached with a physician on the day of testing. The exposures assessed included duration exclusively breastfed, type and duration of supplementation with formula feeding, a diagnosis of wheezy bronchitis before two years of age, systemic antibiotics given before age two, a history of maternal smoking during pregnancy, and exposure to domestic passive smoking in the first year of life.

 Parental atopic disease was defined as symptoms of hay fever and/or asthma in at least one parent when exposed to allergens encountered in the standard testing panel described below. This method is comparable with the one used in the ECRHS [14]. Symptoms among participants were assessed with the questions: “have you ever had hay fever?”, “have you ever had episodes of runny or stuffy nose or sneezing when not having a cold?”, “have you ever had asthma?”, and “have you ever had episodes of wheezing with shortness of breath?” This information was obtained on the day of examination by interview with a physician. Consensus was reached with the accompanying parent. Clinical disease was defined as being present when a positive answer was provided to at least one of the two questions on hay fever and/or the two questions on asthma. 

### 2.3. Serum Total IgE and Skin Prick Tests

Levels of serum total IgE were measured with an enzyme-linked immunosorbent assay (ELISA, on Immulite 2500, DPC, New York, USA). Results were expressed as KIU/L. Values above 150 KIU/L were regarded as elevated. 

 Skin prick tests (SPT) were performed using standard dilutions of ten common aeroallergens. The allergens used were birch, grass, mugwort, horse, dog, cat, house dust mite (*Dermatophagoides pteronyssinus* and *Dermatophagoides farinae*), and two moulds (*Altanaria iridis* and *Cladosporium herbarium*) with positive and negative references being histamine 10 mg/mL in 50% glycerol and glycerol 50%, respectively. The concentrations of allergens were 10 HEP (Soluprick SQ system, ALK Albelló, Denmark). Reactions were read after 15 min. A positive test result was defined as a positive reaction to at least one of the allergens. The reaction to each of the allergens was regarded as positive if the mean wheal diameter ((*D*1 + *D*2) × 1/2) was at least 3 mm. The participants were told to stop using medications that contained antihistamine at least three days before skin testing. 

### 2.4. Bronchial Responsiveness Test

The method of Yan et al. was used for measuring airway responsiveness to inhaled histamine [15]. Each aerosol was inhaled starting with saline and followed by increasing doses of histamine until a cumulative dose of 7.8 *μ*mol had been reached. The test was terminated when the maximum concentration had been reached or when a 20% decline in FEV_1_ had occurred before the end of the dosing regimen. For all subjects experiencing at least a 20% decline in FEV_1_ the concentration causing a 20% fall in FEV_1_ (PD_20_) was calculated. A positive test result (AHR) was defined as a PD_20_ below 3.9 *μ*mol. 

### 2.5. Statistical Analysis

Data were analysed with the statistical package SPSS 16.0 (SPSS, Inc., Chicago, IL, USA). Population characteristics were compared using the unpaired *t*-test for numerical data and the chi-square test for categorical data. To determine the impact of different factors on serum total IgE, we first used univariate linear regression analysis and then multivariate linear regression analysis. The response variable was serum total IgE and the explanatory variables were different perinatal factors, early life factors, socioeconomic factors, demographic factors, clinical, and paraclinical factors. A stipulation of the linear regression analysis is that for each value of the explanatory variable, the values of the response variable should approximately follow the normal distribution. The IgE values in our population did not follow the normal distribution, so we used log (IgE) as the response variable as these data were approximately following the normal distribution. Under the assumption of constant variance, all factors examined were analysed by univariate regression. Based on the univariate analysis, factors that had a relation to serum total IgE that was statistically significant or approaching significance were included in the multivariate analysis. Factors of which we lacked knowledge in a great number of the participating subjects were excluded from the multivariate regression analysis. As a consequence, the multivariate analysis included the factors: birth weight, birth length, smoking in pregnancy, parental disposition to allergy, sex, age, smoking, FEV/FVC, airway hyperresponsiveness, asthma, hay fever, positive skin prick test, and atopic dermatitis. Subjects, on which we did not have information about all of these factors, were excluded from the multivariate analysis. A total of 378 subjects were included in the final model. 

## 3. Results

**Figure 1:**
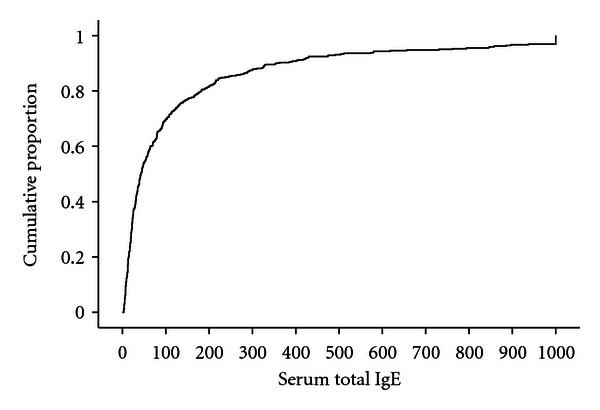


Serum total IgE measurements were available for 421 children. The median value of serum total IgE in the population was 41.8 (Figure 1). In girls, the median value was 33.8, range (2.3–5440), whereas in the boys the median value was 57.4, range (2.1–2173). The geometric mean serum total IgE in the population was 49.3; 60.4 in boys and 41.0 in girls, *P* < .05. The proportion of subjects having elevated serum total IgE, asthma, hay fever, atopic dermatitis, and positive skin prick test was not significantly different between boys and girls (Table 1).

 The results of the univariate analyses are shown in Table 2. The following factors were significantly associated with serum total IgE: parental allergic predisposition, early life wheezy bronchitis, male sex, airway hyperresponsiveness, asthma, hay fever, positive skin prick test, and atopic dermatitis. After multivariate adjustment positive skin prick test, airway hyperresponsiveness, atopic dermatitis, and parental predisposition remained significant predictors of serum total IgE (Table 3). 

## 4. Discussion

This study showed that positive skin prick test, airway hyperresponsiveness, atopic dermatitis, and parental predisposition to atopic disease were significant predictors of serum total IgE in children, 7–17 years of age. In contrast to some previous studies [1–3, 6, 9] we did not find any effect of smoking on serum total IgE. However, this is consistent with the findings in a study by Sherril et al. that found an effect of smoking on IgE only among subjects older than 20 years of age [4]. The association between serum total IgE and gender that previous studies have found in adults was not found in this study of children. Also, a decreasing tendency for IgE with age [2, 5, 7] was not significant in this study, but a comparison with earlier studies is difficult as these mostly included older age groups and a broader range of ages. The association of atopic disease and positive skin prick test to serum total IgE level is consistent with the results of earlier studies [4, 7, 9, 10]. On the contrary we did not find a significant association between asthma and serum total IgE but this was probably due to a significant effect of airway hyperresponsiveness and a strong correlation between this variable and asthma.

 The participation rate in this study was quite low. We tried to address this possible bias by making telephone interviews with a random sample of nonparticipants. This showed a comparable distribution of nonparticipants and participants with respect to sex and age but a slightly lower prevalence of hay fever among nonparticipants. A possible parental recall bias cannot be precluded as parents of atopic children may be more aware of factors that could have influenced the development of the disease [16]. Particularly, parents with confirmed or suspected atopic disease may change behaviour during pregnancy or in early life of the child to try to avoid passing on the disorder [17]. Parental predisposition in this study was defined as symptoms of asthma and/or hay fever in at least one parent when exposed to allergens. This may not be an entirely genetic predisposition as social and environmental factors causing or contributing to the parents' disease could be shared with the child. 

 In conclusion, these results show that serum total IgE in children is significantly associated with positive skin prick test, parental predisposition to atopic disease, airway hyperresponsiveness, and atopic dermatitis. 

## Figures and Tables

**Table 1:** Population characteristics.

	Boys	Girls	Total	*P*-value
	*N* = 222	*N* = 258	*N* = 480	
Age	11.8	12.5	12.2	.004
Serum total IgE	60.4	41.0	49.3	.008
High IgE	53 (26.4)	44 (20.0)	97 (23.0)	.121
Asthma	20 (9.0)	24 (9.3)	44 (9.2)	.912
Hay fever	54 (24.3)	58 (22.5)	112 (23.3)	.634
Atopic dermatitis	57 (25.7)	74 (28.7)	131 (27.3)	.461
Positive skin prick test	50 (22.9)	41 (16.3)	91 (19.4)	.068

**Table 2:** Univariate relationships between different factors and serum total IgE in a sample of children.

Variable	B coefficient	*P*-value
Perinatal factor		
Birth weight (per 100 g)	0.103	.063
Birth length (per cm)	0.018	.125
Gestation (per week)	0.000	.967
Preterm	−0.002	.989
Not breastfed	−0.019	.908
Supplementation	0.001	.991
Smoking in pregnancy	0.066	.184
Smoking in home	−0.056	.382
Parental predisposition	0.133	.013
Season of birth		.926

Early life factors		
Pneumonia	0.128	.117
Antibiotics	−0.006	.923
Wheezy bronchitis	0.319	.0004

Socioeconomic factors		
Mother age	−0.004	.509
Mother education	0.096	.184
Mother alone	−0.001	.984
Mother smoke	−0.107	.130
Father age	−0.010	.110
Father education	0.078	.361
Father smoke	0.006	.941
Household income	0.012	.539

Demographic factors		
Sex	0.168	.008
Age (per year)	−0.018	.101
BMI (per unit)	0.006	.532
Smoking	−0.217	.062

Clinical and paraclinical factors		
FEV/FVC	−0.825	.130
AHR	0.413	.000002
Asthma	0.279	.011
Hay fever	0.387	.000000
Positive skin prick test	0.761	.000000
Atopisk dermatitis	0.264	.0002

**Table 3:** Significant predictors of serum total IgE in a random sample of children, 7–17 years of age.

Variable	B coefficient	Standard error	*P*-value
Positive skin prick test	0.663	0.078	<.001
Airway hyperresponsiveness	0.248	0.084	.003
Atopic dermatitis	0.133	0.066	.046
Parental predisposition	0.100	0.050	.047
